# Effective Phytosanitary Treatment for Export of Oriental Melons (*Cucumis melo* var L.) Using Ethyl Formate and Modified Atmosphere Packaging to Control *Trialeurodes vaporariorum* (Hemiptera: Aleyrodidae)

**DOI:** 10.3390/insects14050442

**Published:** 2023-05-08

**Authors:** Kyeongnam Kim, Dongbin Kim, Tae Hyung Kwon, Byung-Ho Lee, Sung-Eun Lee

**Affiliations:** 1 Institute of Quality and Safety Evaluation of Agricultural Products, Kyungpook National University, Daegu 41566, Republic of Korea; kn1188@knu.ac.kr (K.K.);; 2 Department of Applied Biosciences, Kyungpook National University, Daegu 41566, Republic of Korea

**Keywords:** fumigation, exported oriental melon, the greenhouse whitefly, modified atmosphere packaging, phytosanitary export

## Abstract

**Simple Summary:**

Ethyl formate (EF) can be a potential alternative to methyl bromide for the disinfestation of the greenhouse whitefly, a quarantine pest that affects the exportation of Oriental melon. The study found that 8 g/m^3^ EF for 2 h at 5 °C could be used as a new phytosanitary treatment for melons (*C. melo*) for export with modified atmosphere packaging (MAP) to disinfest the greenhouse whitefly.

**Abstract:**

*Trialeurodes vaporariorum* (Hemiptera: Aleyrodidae), commonly known as greenhouse whitefly, is one of the main insect pests of Oriental melon (*Cucumis melo* var L.) in South Korea. *T. vaporariorum* is of concern as a quarantine pest for the exportation of *C. melo* in Southeast Asian countries. Due to future restrictions on the use of methyl bromide (MB) during quarantine, ethyl formate (EF) represents a potential alternative. In this study, we evaluated EF for its efficacy (probit-9 values) in enabling the export of Oriental melons. The probit-9 value of EF for controlling *T. vaporariorum* was 3.02 g·h/m^3^ after 2 h of fumigation. We also assessed the phytotoxicity of EF on melons when using modified atmosphere packaging (MAP) under low-temperature conditions, which is required for export and trade, to extend shelf-life. In scaled-up trials, we found 8 g/m^3^ EF for 2 h at 5 °C to be suitable as a new phytosanitary treatment against greenhouse whitefly for exported Oriental melons when using MAP. No phytotoxic damage was found 28 d after fumigation at 5 °C in terms of five quality parameters (firmness, sugar content, mass loss, color change, and external damage).

## 1. Introduction

Recently, the demand from China for fresh fruits and vegetables from neighboring countries has increased. From 2008 to 2017, the demand from China increased 5-fold, with the main exporting countries being Vietnam, Laos, Thailand, and Myanmar [1]. Myanmar, in particular, exported 874,127 tons of melons, including watermelons, honeydew melons, and cantaloupes, between 2017 and 2018 [1].

Oriental melons (*Cucumis melo* var L.) are cultivated mainly in South Korea and represent one of the most important summer agricultural crops in the country. Due to their unique appearance and sweet taste, they are exported to other countries, including Southeast Asian countries [2]. More than 70% of Oriental melons are harvested in Seongju-gun and surrounding areas in Kyungpook province, South Korea, and are exported to Japan, Hong Kong, Taiwan, and Malaysia. Oriental melon exports from South Korea reached 215, 436, and 415 tonnes in 2018, 2019, and 2020, respectively [3,4].

During melon cultivation, *Trialeurodes vaporariorum* (Hemiptera: Aleyrodidae), greenhouse whitefly, spreads viruses and causes serious damage to melons by sucking the plant sap. Therefore, *T. vaporariorum* causes a steep reduction in melon yield and leads to a decreased market value due to poor melon quality [5,6]. *T. vaporariorum* is a critical agricultural insect pest in terms of both plantation and crop protection worldwide [7,8]. This insect pest is classified as a quarantine pest, and disinfestation is required for the trade of host plants in some regions, including Europe, Japan, and South Asia [9,10,11,12]. Although *T. vaporariorum* is not a quarantine pest in South Korea and only causes damage during crop cultivation, quarantine issues may soon arise in some melon-importing countries. Therefore, phytosanitary disinfestation studies are urgently needed, and quality control of *C. melo* by post-phytosanitary treatment during the export of melons is required, which is an important factor in promoting the exportation of these fruits. The scientific outcomes of these studies will be used to find a replacement for methyl bromide (MB) fumigation, which has restricted use. Strong recommendations to use technically feasible alternatives have been made by the Food and Agriculture Organization (FAO) and International Plant Protection Convention [13].

Low-temperature storage (5 °C) is necessary to avoid softening, browning, and overall decay of melons during export. Moreover, modified atmosphere packaging (MAP), which increases CO_2_ and decreases O_2_ concentrations, while also reducing respiration and moisture, is required. These conditions are important for maintaining the quality of melons during export [3,14,15]. Phytosanitary disinfestation guidelines for the control of *T. vaporariorum* and quality control of *C. melo* may affect the export of melons from South Korea.

The most common and economical phytosanitary treatment of melons is based on MB fumigation, which needs to be replaced with other options because of its ozone-depleting properties, worker safety hazards, and residue issues [16,17]. Recently, ethyl formate (EF) fumigation has been developed and used as an MB alternative for fruits, vegetables, nursery plants, and non-food commodities [16,17,18,19,20,21,22,23,24]. Due to the potential benefits of using EF, including safer working environments and compliance with residue-free regulations, the aims of this study were (1) to investigate the efficacy of EF fumigation on *T. vaporariorum* control in terms of satisfying the International Plant Protection Convention (IPPC) requirements; (2) to evaluate the quality of melons for export under export environments, such as low temperature and MAP packing conditions; and (3) to conduct commercial trials to determine whether EF application is technically feasible in practice.

## 2. Materials and Methods

### 2.1. Insects and Chemicals

*Trialeurodes vaporariorum* was collected from a *C. melo* plantation in the Seongju-gun area, South Korea in 2021. *T. vaporariorum* was reared in the lab (Kyungpook National University, Daegu, South Korea) on *Nicotiana tabacum* (L.) and *Solanum melongena* (L.) as host plants and kept at 24 °C ± 1 °C with 60–70% relative humidity with a photoperiod of 16:8 (L:D) h. EF (fumate, 99% purity) was supplied by SAFEFUME (Hoengseong-gun, South Korea).

### 2.2. Efficacy of EF on T. vaporariorum in Lab Conditions

The efficacy of EF was evaluated against adults of *T. vaporariorum*. More than 30 individuals in the adult stage were added to a breeding dish (diameter = 4.5 cm) with *N. tabacum* leaves. These adult insects were fumigated at 5 °C for 2 h with 0.25, 0.5, 1, 2, 3, and 4 g/m^3^ of EF. The flies were taken to an insect-rearing room after fumigation. The mortality of the fumigated *T. vaporariorum* was assessed by visually inspecting movement using a microscope (Stemi 305, Carl Zeiss Suzhou Co., Ltd., Suzhou, China) at three days after fumigation. All treatments and controls were replicated three times. The temperatures were recorded by data loggers (Thermo Recorder TR-72U; T&D Corp., Matsumoto, Japan). Desiccators with a volume of 6.8 L were used as fumigation chambers. The desiccators were sealed with glass stoppers equipped with a septum (Cat. No. 15419; Alltech Associates Australia, Sydney, Australia) and tightly sealed using high-vacuum grease (Dow Corning, Midland, MI, USA). A piece of filter paper (Whatman No. 1) was inserted into the glass stopper to allow liquid EF to evaporate efficiently in the desiccator. A magnetic bar was placed at the bottom of the desiccator to stir the contents. The doses of fumigant and concentration × time (Ct) products were calculated according to a previously described method [25].

### 2.3. Assessments of Sorption and Phytotoxicity of EF; Preliminary Tests with/without MAP

The sorption rate of EF on *C. melo* was determined preliminarily by treating the melons with EF at a 10% (*w*/*v*) *C. melo* loading ratio (Lr) in a 0.275 m^3^ (0.5 × 0.4 × 1.3 m) fumigation chamber. EF concentration in the fumigation chamber was sampled at 0 (C_0_, initial time), 1, and 2 h. The sorption rate was calculated as [1 − (C/C_0_)] × 100. The phytotoxicity of EF with regard to firmness, sugar content, mass loss, color change, and overall decay of *C. melo* after 2 h of EF fumigation at a concentration of 15 g/m^3^ was evaluated after four weeks. The phytotoxic assessment was conducted for *C. melo* with and without MAP after EF fumigation. MAP is a method of maintaining CO_2_ at about 5–10% during storage by applying permeable materials containing 5% low-density polyethylene (LDPE) zeolite [3]. Firmness (kgf) was measured using a fruit firmness tester (53205 Digital fruit firmness tester, TR Turoni, Forli, Italy) equipped with an 8 mm steel plunger. Firmness was measured three times for each fruit, and five fruits were measured for each treatment. The sugar content (Brix, %) was measured using a portable refractometer (PAL-1, Atago Co., Ltd., Tokyo, Japan). Briefly, the fruits were cut into sections, and 0.5 mL of the juice was dropped onto the portable refractometer. Five fruits were measured for each treatment. The color change was measured using a colorimeter (TES-135A, TES Electrical Electronic Corp., Taipei, Taiwan) that expressed color as Hunter L*, a*, and b* values. The mass loss was expressed as the ratio between the mass before treatment and after a certain storage period. External damage or overall decay was evaluated by scoring *C. melo* as zero (not affected), one (slight, <5% affected), two (bad, <50% affected), or three (severe, >50% affected) and photographing.

### 2.4. Commercial Trials

Commercial trials of *C. melo* for export were carried out in a 10 m^3^ fumigation chamber located at the Animal & Plant Quarantine Agency (APQA, Gimcheon, South Korea). The fumigation chamber was filled with 5% *C. melo* Lr. *T. vaporariorum* was added to insect breeding dishes (diameter: 9 cm) and placed both inside and outside the boxes to check the permeability of EF gas into the boxes, which are currently used for commercial and international trading in South Korea. Gas sampling lines were set at three levels (top, middle, and bottom) in the fumigation chamber to check the EF concentration. Gas sampling was performed using a 1 L Tedlar bag (SKC Inc., Dorset, UK) with vacuum pump (Gast Manufacturing Inc., Benton Harbor, MI, USA) at hourly intervals (0, 1, and 2 h), and the EF concentration was measured using gas chromatography with flame-ionization detection (GC-FID). In the commercial trials, 8 g/m^3^ EF was added to a fumigation chamber using a vaporizer for 2 h at 5 °C ± 1 °C. The EF concentration in the sampled gas was analyzed by GC-FID. After 2 h of fumigation, the container was opened and ventilated for 2 h to decrease the gas concentration, and the desorption rate was measured using GC-FID. The *C. melo* fruits were then transported to a storage room for phytotoxic assessment. The mortality rate of the adult *T. vaporariorum* was evaluated using a microscope after fumigation for 3 d. The phytotoxic effects on *C. melo* were evaluated for 28 d.

### 2.5. Statistical Analysis

The toxicological dose response of *T. vaporariorum* to EF was analyzed on the basis of a probit analysis [26]. The indices of toxicity derived from this analysis were L(Ct)_50_ (median lethal concentration that causes 50% response (mortality)) and L(Ct)_99_ (median lethal concentration that causes 99% response (mortality)) in exposed *T. vaporariorum*. These indices were determined from a range of at least 10 different Ct products to ensure that the observed data covered 0 to 100% mortality and adequately covered the intermediate range. To analyze the phytotoxic damage caused to *C. melo* by EF fumigation, one-way ANOVA was performed based on Tukey’s Studentized range (honest significant difference test) at *p* = 0.05 using SAS (ver. 9.4; SAS Institute Inc., Cary, NC, USA).

## 3. Results

### 3.1. Efficacy of EF on T. vaporariorum under Lab Conditions

The efficacy of EF at concentrations ranging from 0.4 to 3.5 g/m^3^ against adult *T. vaporariorum* was investigated. A fumigated *T. vaporariorum* individual was considered to be dead when no movement could be observed under a microscope. The L(Ct)_50_ and L(Ct)_99_ values for EF activity against *T. vaporariorum* adults were 0.79 and 2.32 g·h/m^3^, respectively, and the probit-9 value was 3.02 g·h/m^3^ with fitted slopes of 4.95 ± 0.4 for 2 h of fumigation at 5 °C (Table 1). We obtained probit-9 values at the 95% confidence level, implying this result could satisfy the requirements of export countries.

### 3.2. Assessments of Sorption and Phytotoxicity of EF Fumigation with/without MAP

**Figure 1:**
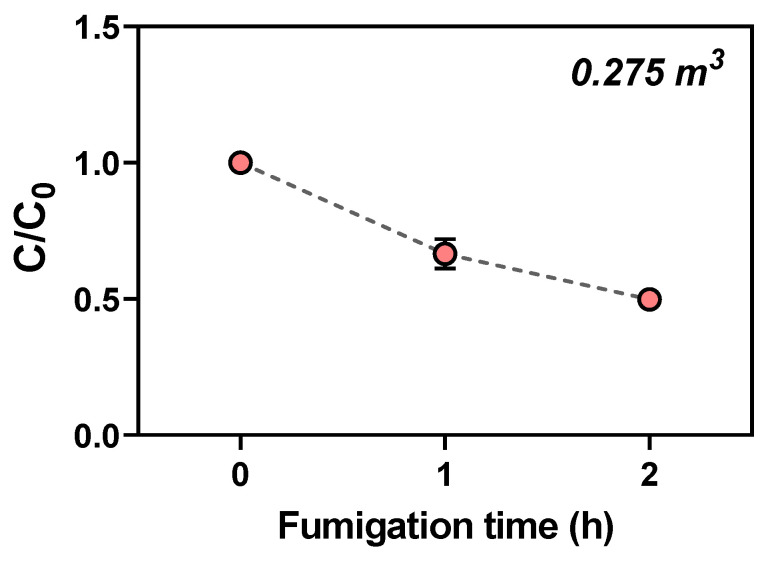
Concentration loss of ethyl formate (EF) (C/C_0_) in a 0.275 m^3^ fumigation chamber with a 10% loading ratio (*w*/*v*) of *Cucumis melo* (EF 15 g/m^3^ for 2 h at 5 °C). C = EF concentration determined at one of the time intervals and C_0_ = initial EF concentration.

In the preliminary test, the concentration of EF in a 0.275 m^3^ fumigation chamber with a 10% loading ratio (Lr) (*w*/*v*) of *C. melo* decreased gradually during 2 h of EF fumigation. The concentration loss rate of EF was approximately 50% after 2 h of EF fumigation (Figure 1) with a Ct product of 11.88 g·h/m^3^, which was higher than the probit-9 value for *T. vaporariorum*.

**Figure 2:**
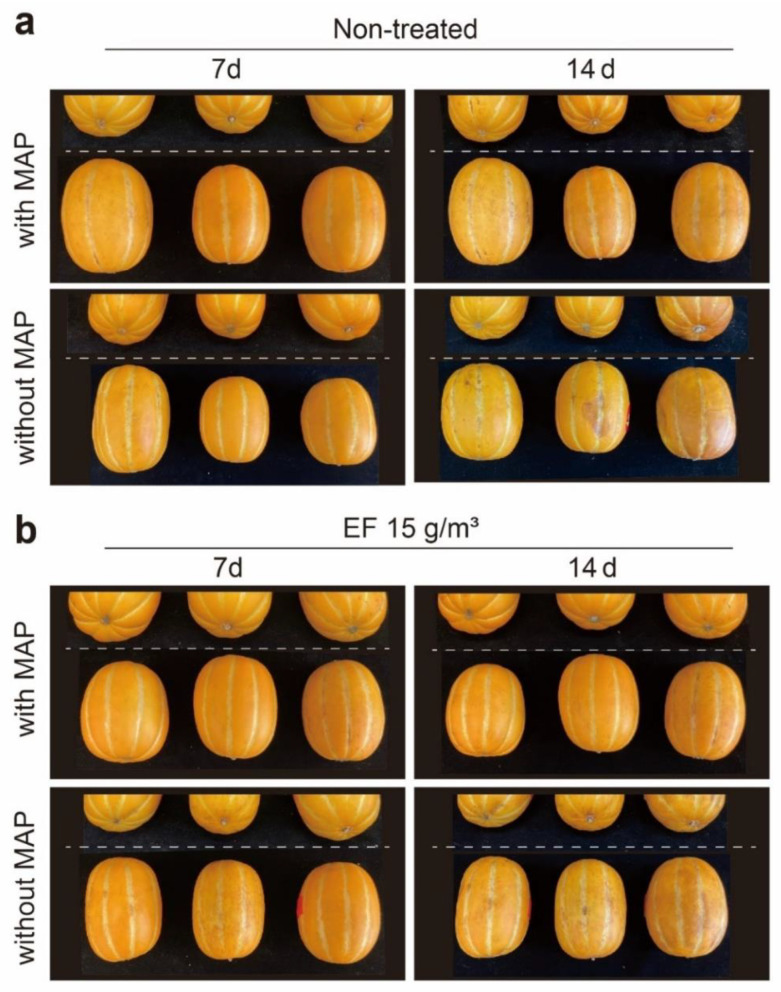
Photographs of *Cucumis melo* at 7 and 14 days after 15 g/m^3^ EF fumigation with or without modified atmosphere packaging (MAP). (**a**) Non-treated group; (**b**) EF fumigation was conducted in a 0.275 m^3^ (0.5 × 0.4 × 1.3 m) fumigation chamber for 2 h at 5 °C.

In the phytotoxic assessments, there was no significant difference in the firmness of *C. melo* among all the treatments after 14 d (*p* > 0.05), as the firmness of EF-untreated groups with/without MAP during 14 d of storage was about 24.0 kgf, while that of EF-treated groups under 11.88 g·h/m^3^ reached 25.0 kgf. However, 28 d after EF fumigation, there was a significant difference between the EF-untreated/treated group without MAP and the EF-untreated/treated groups with MAP with regard to firmness, as the EF-untreated/treated group with MAP during 28 d of storage exhibited firmness in the range from 20.0 to 23.0 kgf, while the EF-untreated/treated group without MAP during 28 d of storage showed a remarkable decrease in the range of 7.7 to 8.5 kgf (Table 2 and Figure 2).

In terms of sugar content, weight loss, and color change after 14 and 28 d, there were no significant differences, regardless of MAP. Moreover, no difference in external damage was observed after 7 d (Figure 2), but *C. melo* scored 2 on the index for external damage in the EF-untreated/treated group without MAP after 14 d (Table 2 and Figure 2).

### 3.3. Commercial Trials

**Figure 3:**
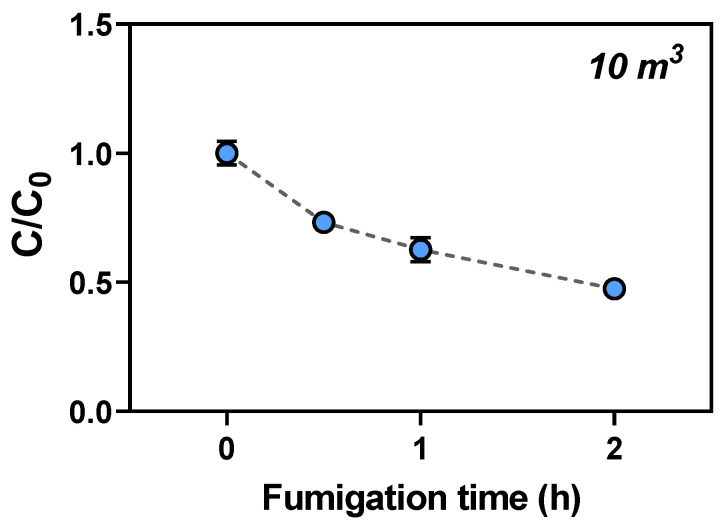
Concentration loss of EF for the commercial trial (C/C_0_) in a 10 m^3^ fumigation chamber with 5% loading ratio (*w*/*v*) of *C. melo* (EF 8 g/m^3^ for 2 h at 5 °C). C = EF concentration determined at one of the time intervals and C_0_ = initial EF concentration.

In the commercial trials, the concentration of EF in a 10 m^3^ fumigation chamber with a 5% Lr (*w*/*v*) of *C. melo* decreased gradually during a 2 h period of fumigation, similar to the preliminary test, as shown in Figure 1. The concentration loss rate of EF in the chamber was approximately 50% after 2 h of EF fumigation (Figure 3) with a Ct product of 8.77 g·h/m^3^, which was higher than the probit-9 value (3.02 g·h/m^3^) for *T. vaporariorum*.

**Figure 4:**
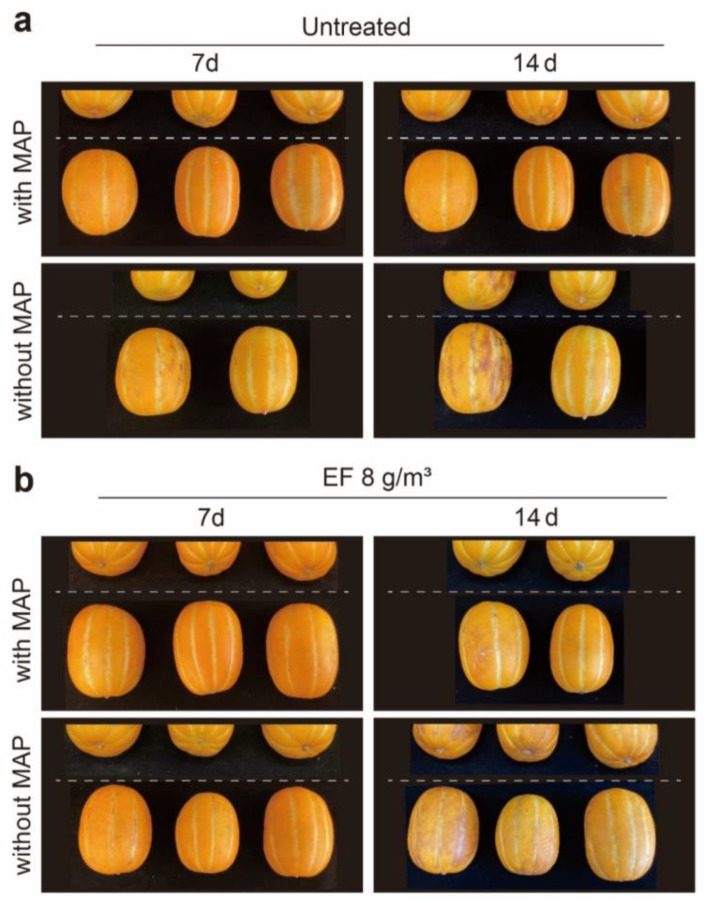
Photographs of *Cucumis melo* at 7 and 14 days after fumigation with 8 g/m^3^ ethyl formate (EF) in commercial trials with or without modified atmosphere packaging (MAP). (**a**) Untreated group; (**b**) EF fumigation conducted in a 10 m^3^ chamber for 2 h at 5 °C.

Under these fumigation conditions, no external damage was observed after 14 d, but *C. melo* scored 2 for external damage in the EF-untreated/treated group without MAP after 28 d (Figure 4 and Table 3). According to the phytotoxicity assessments, there was no significant difference in the firmness of *C. melo* among all treatments after 14 d (*p* > 0.05), as the firmness of the EF-untreated groups with/without MAP during 14 d of storage was about 23.0–24.0 kgf, while that of groups treated with 8.77 g·h/m^3^ EF reached 21.0–24.5 kgf (Table 3).

After 28 d, there was a significant difference between the EF-untreated/treated groups with and without MAP in terms of firmness (Table 3). The EF-untreated/treated group with MAP exhibited firmness in the range of 21.0 to 24.5 kgf, while the group without MAP showed a remarkable decrease in the range of 7.1 to 8.1 kgf, over 28 d of storage (Table 3). Regardless of the MAP, 28 d after EF fumigation, there were no significant differences in sugar content or mass loss and no significant difference in color change after 14 and 28 d. The factor causing phytotoxic damage to *C. melo* was the presence or absence of MAP, not EF itself. Therefore, MAP must be used for long-term storage after EF treatment. EF treatment did not affect the quality of *C. melo*.

## 4. Discussion

### 4.1. Fumigation of T. vaporariorum Using EF

EF fumigation has been considered primarily in the field of quarantine as an alternative to MB fumigants [16,17]. Phosphine (PH_3_), which is in the limelight as another MB alternative, has raised doubts about its use because of the emergence of resistance in stored insect pests [27,28]; accordingly, the combined use of PH_3_ and EF is being studied [29]. Another disadvantage of using PH_3_ is the long duration of fumigation required, whereas a shorter treatment period is needed when using EF. In addition to these points, EF is a natural product that occurs in nature and is produced mainly by plants and microorganisms [20,30,31]. Therefore, it is generally recognized as a safe flavoring ingredient and is currently used in the food and cosmetic industries. Interestingly, some researchers have tried to develop metabolically engineered microorganisms to produce larger amounts of EF to avoid the requirement for chemical synthesis [32]. For example, EF, propionic acid, and acetaldehyde are produced at high levels during citric fermentation by *Aeromonas salmonicida* [32].

With these points in mind, EF can be considered for use in closed systems such as vinyl houses and smart farms to control agricultural insect pests [33]. Presently, various insect pests are controlled by EF fumigation in vinyl houses, and EF fumigation in an Oriental melon vinyl house to control *Bemisia tabaci* has been studied [33]. The L(Ct)_50_ and L(Ct)_90_ values for EF fumigation against *B. tabaci* were found to be 0.41 and 1.67 g·h/m^3^, respectively, for 2 h of exposure at 29.0 °C [33]. When the period of exposure to fumigation was increased to 4 h and 12 h, the L(Ct)_50_ and L(Ct)_90_ values also increased [33]. According to these results, the authors suggested that a 2 h exposure to EF fumigation is suitable to control agricultural insect pests in vinyl houses. EF fumigation is not suitable for glasshouses, because of leaking gas, but gas leaks do not occur from vinyl houses. Currently, EF fumigation is suitable for application in vinyl houses. 

Other studies of EF fumigation against quarantine insect pests have determined that EF is effective against *Aphis gossypii* and *Myzus persicae*, with L(Ct)_99_ values of 4.44 and 7.66 g·h/m^3^, respectively, for 2 h of fumigation at 5 °C [29,34]. In the present study, *T. vaporariorum* was controlled by 2 h of EF fumigation at 5 °C, obtaining L(Ct)_50_ and L(Ct)_99_ values of 0.79 and 2.32 g·h/m^3^, respectively, and thus *T. vaporariorum* has been confirmed to be more sensitive to EF compared to the reported efficacy of EF against other insect pests under the same fumigation conditions.

### 4.2. Phytotoxicity of EF and Its Efficacy against Pests of Agricultural Fruits

Oriental melons have previously been used to evaluate phytotoxicity during EF fumigation in a vinyl house [33]. The results revealed that Oriental melons did not show phytotoxic symptoms at different developmental stages under different periods of exposure (2 h, 4 h, and 12 h) to EF fumigation. Kwon et al. (2022) showed 100% control of *B. tabaci* under all experimental conditions, and the researchers observed no phytotoxic effects on yellow melon using EF fumigation at a concentration of 2 g/m^3^ for 2 h [33]. However, the authors found severe damage to the shoots of red pepper as a comparable plant at higher humidity and over longer periods of fumigation.

Simpson et al. (2004) showed that EF fumigation of strawberries at treatment concentrations in the range of 0.8 (equivalent to 24 g/m^3^) to 2.4% (equivalent to 72 g/m^3^) exhibited complete mortality against western flower thrips (*Frankliniella occidentalis*), but not two-spotted spider mites (*Tetranychus urticae*), with 60% of mortality under 2.4% EF fumigation [20]. No significant difference was found in the EF-treated strawberries when compared with the untreated fruits [20]. They did find some differences in volatile compound production, including increased levels of acetaldehyde, ethanol, and ethyl acetate, in EF-treated strawberries.

In a recent study by Jeon et al. (2022), *Drosophila suzukii*, which is well known as a quarantine pest that spreads rapidly in berries, was used to examine the synergistic effect of combined treatment with EF and cold temperatures at 1 °C and 5 °C on imported grapes [35]. They found that, at lower temperatures, all stages of *D. suzukii* were effectively controlled by EF fumigation. The authors determined a high sorption rate (24%) of EF after 4 h of fumigation that was at 15% of the grape loading ratio in a 0.65 m^3^ fumigation chamber. Consequently, there was an increase in *D. suzukii* mortality with a decreased grape loading ratio in the chamber. *D. suzukii* was completely controlled for 7 days after EF fumigation at a lower temperature with no symptoms of phytotoxicity in the grapes.

The production quality of certain crops and fruits may be damaged by EF fumigation. Therefore, each plant species needs to be examined for its reaction to EF fumigation for agricultural insect pests.

### 4.3. Packaging of C. melo with or without EF Fumigation

Modified atmosphere packaging of Oriental melons has been well studied, as they need specific packaging during export and storage because of their short shelf-life and susceptibility to cold-induced damage [14]. Vessel transportation is readily selected for the export of Oriental melons because of the low cost of transportation, but it takes 3–10 days longer than air transportation, leading to increased decay [36]. Using polyethylene (PE) film and X-tend modified atmosphere (MA)/modified humidity film for Oriental melons at 4 °C and 10 °C for 14 days was found to prolong the shelf-life and reduce the browning of the fruit during storage [14].

Ethyl formate fumigation in combination with the MAP system in our study showed that there were no phytotoxic effects on Oriental melons, with complete control of *T. vaporariorum*. As our results showed no changes in five important parameters for quality control, including firmness, sugar content, mass loss, color change, and external damage, the combination of MAP with EF fumigation is a very useful method for reaching the requirements of importing countries for Oriental melons.

## 5. Conclusions

During the export of Oriental melon, EF was investigated as a fumigant to control *T. vaporariorum* to meet the standards of several countries that have designated *T. vaporariorum* as a quarantine pest. We assessed the use of EF as an alternative to MB in terms of its potential phytotoxic effects on Oriental melons and its efficacy against *T. vaporariorum*. The probit-9 value of EF was found to be 3.02 g·h/m^3^, which was lower than the L(Ct) product value. This study proved the suitability of EF as an alternative to MB. In the future, it will be necessary to examine fumigation with EF in more crops to eradicate insect pests in agricultural industries.

## Figures and Tables

**Table 1:** L(Ct) (lethal concentration × time) and probit-9 values following *Trialeurodes vaporariorum* exposure to ethyl formate (EF) for 2 h.

Temp. (°C)	Stage	^1^ L(Ct)_50_ (g·h/m^3^, 95% CL ^2^)	L(Ct)_99_ (g·h/m^3^, 95% CL)	Probit-9 (g·h/m^3^)	Slope ± SE	df	χ^2^
5	Adult	0.79 (0.73–0.84)	2.32 (2.01–2.82)	3.02 (2.84–3.23)	4.95 ± 0.4	10	19.22

^1^ L(Ct)_50_ = median lethal concentration that causes 50% response (mortality); L(Ct)_99_ = lethal concentration that causes 99% response (mortality); ^2^ CL: confidence level

**Table 2:** Phytotoxic assessment of *Cucumis melo* fumigated with 15 g/m^3^ EF for 2 h at 5 ± 1 °C.

EF Dosage (g/m^3^) with or without MAP ^1^	Fumigation Time (h)	Ct Products (g·h/m^3^)	Storage Period (d)	Firmness (kgf, Mean ± SE)	Sugar Content (Brix, %, Mean ± SE)	Weight Loss (Mean ± SE)	Color Change (Mean ± SE)	External Damage ^2^
Untreated with MAP	-	-	14	23.97 ± 0.15 ^a3^	12.86 ± 1.57 ^a^	5.08 ± 0.56 ^a^	119.45 ± 2.51 ^a^	0
Untreated without MAP				23.53 ± 0.73 ^a^	13.63 ± 1.36 ^a^	6.73 ± 0.61 ^a^	119.69 ± 4.49 ^a^	2
EF 15 g/m^3^ with MAP	2	11.88		25.40 ± 0.60 ^a^	15.76 ± 1.18 ^a^	5.16 ± 0.44 ^a^	117.00 ± 1.52 ^a^	0
EF 15 g/m^3^ without MAP				25.03 ± 0.58 ^a^	14.20 ± 1.07 ^a^	6.94 ± 0.50 ^a^	115.08 ± 3.04 ^a^	2
Untreated with MAP	-	-	28	20.05 ± 0.05 ^a^	16.33 ± 3.28 ^a^	11.41 ± 1.19 ^a^	113.65 ± 1.35 ^a^	0
Untreated without MAP				7.7 ± 2.28 ^b^	15.33 ± 2.13 ^a^	15.77 ± 1.53 ^a^	108.62 ± 6.48 ^a^	2
EF 15 g/m^3^ with MAP	2	11.88		22.89 ± 1.84 ^a^	16.43 ± 4.86 ^a^	13.60 ± 0.71 ^a^	112.64 ± 1.74 ^a^	0
EF 15 g/m^3^ without MAP				8.52 ± 0.95 ^b^	16.73 ± 1.20 ^a^	14.03 ± 1.04 ^a^	99.13 ± 3.00 ^a^	2

^1^ MAP: modified atmosphere packaging; ^2^ damage index: 0 (none), 1 (slight), 2 (bad), 3 (severe); ^3^ means in a column followed by the same letter are not significantly different at the 5% level (a > b).

**Table 3:** Commercial trials and phytotoxic assessment of *Cucumis melo* fumigated with 8 g/m^3^ of EF for 2 h at 5 ± 1 °C.

EF Dosage (g/m^3^) with or without MAP ^1^	Fumigation Time (h)	Ct Products (g·h/m^3^)	Storage Period (d)	Firmness (kgf, Mean ± SE)	Sugar Content (Brix, %, Mean ± SE)	Weight Loss (Mean ± SE)	Color Change (Mean ± SE)	External Damage ^2^
Untreated with MAP	-	-	14	24.11 ± 0.14 ^a3^	13.87 ± 1.44 ^a^	4.79 ± 0.15 ^a^	121.40 ± 1.41 ^a^	0
Untreated without MAP				24.53 ± 0.66 ^a^	13.15 ± 0.36 ^a^	5.73 ± 0.71 ^a^	119.23 ± 3.40 ^a^	2
EF 8 g/m^3^ with MAP	2	8.77		23.40 ± 0.31 ^a^	14.76 ± 1.09 ^a^	5.36 ± 0.54 ^a^	117.46 ± 2.51 ^a^	0
EF 8 g/m^3^ without MAP				24.03 ± 0.19 ^a^	13.20 ± 2.07 ^a^	6.51 ± 0.40 ^a^	116.18 ± 2.84 ^a^	2
Untreated with MAP	-	-	28	21.15 ± 0.25 ^a^	15.33 ± 3.10 ^a^	12.44 ± 1.40 ^a^	114.60 ± 1.90 ^a^	0
Untreated without MAP				7.1 ± 1.22 b ^b^	15.89 ± 1.13 ^a^	13.70 ± 1.61 ^a^	118.60 ± 3.40 ^a^	2
EF 8 g/m^3^ with MAP	2	8.77		24.49 ± 0.94 ^a^	15.87 ± 3.61 ^a^	13.13 ± 1.70 ^a^	113.61 ± 2.70 ^a^	0
EF 8 g/m^3^ without MAP				8.12 ± 1.91 ^b^	16.12 ± 0.91 ^a^	14.24 ± 1.21 ^a^	110.33 ± 2.08 ^a^	2

^1^ MAP: modified atmosphere packaging; ^2^ damage index: 0 (none), 1 (slight), 2 (bad), 3 (severe); ^3^ means in a column followed by the same letter are not significantly different at the 5% level (a > b).

## Data Availability

Data are contained within the article.
